# Correction: Sonodynamic therapy induces oxidative stress, DNA damage and apoptosis in glioma cells

**DOI:** 10.1039/d1ra90118d

**Published:** 2021-06-01

**Authors:** Yue Sun, Haiping Wang, Kun Zhang, Jingfei Liu, Pan Wang, Xiaobing Wang, Quanhong Liu

**Affiliations:** National Engineering Laboratory for Resource Development of Endangered Crude Drugs in Northwest China, The Key Laboratory of Medicinal Resources and Natural Pharmaceutical Chemistry, The Ministry of Education, College of Life Sciences, Shaanxi Normal University Xi’an Shaanxi 710119 People’s Republic of China lshaof@snnu.edu.cn +86-29-85310275

## Abstract

Correction for ‘Sonodynamic therapy induces oxidative stress, DNA damage and apoptosis in glioma cells’ by Yue Sun *et al.*, *RSC Adv.*, 2018, **8**, 36245–36256, DOI: 10.1039/C8RA07099G.

The authors regret that there was an error in Fig. 7 in the original article. The DVDMS and SDT + NAC groups were misplaced in the original figure. The correct figure is given here. The results and conclusions reported here are unaffected by this change.

**Fig. 7:**
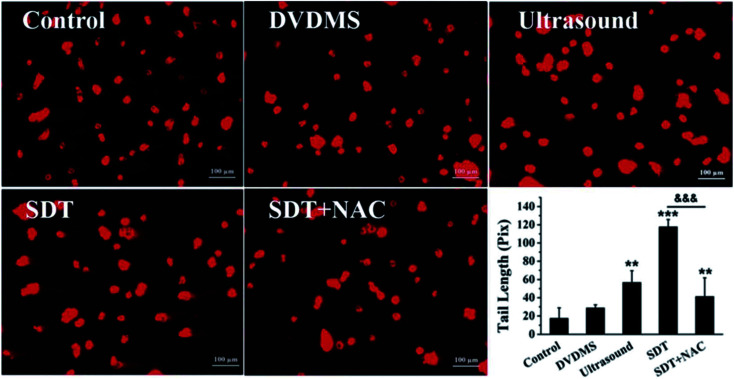
Examination of DNA damage by comet assay in U373 cells. The DNA damage was evaluated by gel electrophoresis and analyzed by CASP software, and the DNA tail length was calculated after different treatments. The data are represented as means ± S.D (*n* = 3). *p < 0.05, **p < 0.01 and ***p < 0.001 *versus* the untreated control, ^&&&^*p* < 0.001 SDT group *versus* SDT + NAC group.

The Royal Society of Chemistry apologises for these errors and any consequent inconvenience to authors and readers.

## Supplementary Material

